# Protective effects of molecular hydrogen on steroid-induced osteonecrosis in rabbits via reducing oxidative stress and apoptosis

**DOI:** 10.1186/s12891-017-1431-6

**Published:** 2017-02-02

**Authors:** Jia Li, Zhaogang Ge, Lihong Fan, Kunzheng Wang

**Affiliations:** 1 Department of Orthopedics, The First Affiliated Hospital of Xi’an Jiaotong University, Yanta West Road, Xi’an, Shaanxi Province 710061 People’s Republic of China; 2 Department of Joint Surgery, Honghui Hospital of Xi’an Jiaotong University, Xi’an, Shaanxi Province 710054 People’s Republic of China; 3 The first department of Orthopedics, The Second Affiliated Hospital of Xi’an Jiaotong University, Xi’an, Shaanxi Province 710004 People’s Republic of China

**Keywords:** Molecular hydrogen, Osteonecrosis, Oxidative injury, Vascular injury, Apoptosis

## Abstract

**Background:**

The objective of this study was to investigate the protective effects of molecular hydrogen, a novel and selective antioxidant, on steroid-induced osteonecrosis (ON) in a rabbit model.

**Methods:**

Sixty rabbits were randomly divided into two groups (model group and hydrogen group). Osteonecrosis was induced according to an established protocol of steroid-induced ON. Rabbits in the hydrogen group were treated with intraperitoneal injections of molecular hydrogen at 10 ml/kg body weight for seven consecutive days. Plasma levels of total cholesterol, triglycerides, soluble thrombomodulin(sTM), glutathione(GSH) and malondialdehyde(MDA) were measured before and after steroid administration. The presence or absence of ON was examined histopathologically. Oxidative injury and vascular injury were assessed in vivo by immunohistochemical staining of 8-hydoxy-2-deoxyguanosine(8-OHdG) and MDA, and ink artery infusion angiography. The terminal deoxynucleotidyl transferase-mediated dUTP nick end labeling (TUNEL) assays were performed to measure apoptosis.

**Results:**

The incidence of steroid-induced ON was significantly lower in hydrogen group (28.6%) than that in model group (68.0%). No statistically differences were observed on the levels of total cholesterol and triglycerides. Oxidative injury, vascular injury and apoptosis were attenuated in the hydrogen group compared with those in the model group in vivo.

**Conclusions:**

These results suggested that molecular hydrogen prevents steroid-induced osteonecrosis in rabbits by suppressing oxidative injury, vascular injury and apoptosis.

## Background

Steroid-induced osteonecrosis(ON), especially at hip joint, is one of the most common complications of corticosteroids. As most of the affected patients are young individuals and the surgical prognosis of steroid-induced ON is generally poor, it is highly desirable to develop a strategy to prevent its occurrence. Hyperlipidemia, hypercoagulable condition, fat emboli, intraosseous pressure rise with fat cell enlargement, enhanced vasoconstriction and vascular endothelial dysfunction after steroid administration have been suggested to cause steroid-induced ON [1–3]. However, the detailed pathophysiological mechanisms of steroid-induced ON remain unclear. It is generally accepted that microcirculatory disturbances within bone underlie the development of steroid-induced ON [4]. To clarify the pathophysiology of steroid-induced ON and develop promising methods to prevent its occurrence, a number of preclinical animal ON models have been established including traumatic ON(surgical vascular deprivation, physical and chemical insult–induced traumatic ON) and non-traumatic ON(spontaneous, steroid-induced or steroid-associated, lipopolysaccharide-induced or their combination-induced, horse serum-induced and alcohol-induced) [5]. Many researchers described steroid-induced ON models in rabbits by administering methylprednisolone intramuscularly, which had an advantage with etiology similar to ON patients but also with disadvantages of only early-stage ON [6–8]. Multifocal ON lesions were identified histologically in the femora, implying the effectiveness of this model for the evaluation of therapeutic efficacy of interventions developed for prevention of steroid-induced ON.

Recently, it was indicated that oxidative stress, which is associated with numerous pathological conditions including vascular injury and cell apoptosis [9, 10], plays a crucial role in the development of steroid-induced ON [11]. More recently, the pro-oxidant buthionine sulphoximine has been proved to successfully induce ON in rats model [12] and the antioxidative substances including vitamin E [13, 14], glutathione (GSH) [11] and edaravone [7] have been demonstrated to be effective in preventing steroid-induced ON. Vascular injury and apoptosis, which can be induced by oxidative stress [10, 11], also participate in the development of steroid-induced ON [15, 16]. Thus, we hypothesized that antioxidants may be effective in preventing the development of steroid-induced ON by blocking the development of oxidative stress, vascular injury and cell apoptosis.

In 2007, Ohsawa et al. [17] found that diatomic hydrogen(H2) could selectively reduce cytotoxic reactive oxygen species(ROS) including hydroxyl radical (•OH) and peroxynitrite(ONOO-), without affecting physiological ROS including H2O2 and the nitric oxide radical (NO•), which have been shown to play important roles in immune defence systems and signal transduction [18–20]. Moreover, in a rat model of ischaemia–reperfusion brain injury, he and his colleagues observed that molecular hydrogen is more effective than edaravone and as effective as FK506 in alleviating oxidative injury, which indicated the potential of molecular hydrogen for therapy. Then, molecular hydrogen was proposed as a novel and selective antioxidant and researches on the potential use of hydrogen as therapeutic agents in various diseases were conducted. It was showed that hydrogen can exert beneficial effects in diverse diseases of animal models related to oxidative stress, including ischaemia–reperfusion injury, radiation complication, atherosclerosis and sepsis [21–24]. Besides, it was reported that administration of H2-saturated water can also improve lipid metabolism [25–27]. Compared with other antioxidants such as vitamin E, vitamin C and edaravone, hydrogen gas can diffuse rapidly into tissue and subcellular compartments including mitochondria and the nucleus, and can further eliminate ROS generated in mitochondria and protect DNA from oxidative damage in nuclear [17]. Accordingly, we assumed that molecular hydrogen might exert beneficial effects on preventing steroid-induced ON by reducing oxidative stress and apoptosis. The aim of this study was to test whether hydrogen can prevent the development of steroid-induced ON and the possible mechanisms involved.

## Methods

### Animal, grouping and treatment

All the animals used were housed at the Experimental Animal Center of Xi’an Jiaotong University, China and were maintained on the standard laboratory diet and water ad libitum. All protocols were approved by the Animal Ethical Committee of the Xi’an Jiaotong University and were followed in accordance with the NIH Guide for the Care and Use of Laboratory Animals.

Sixty mature male New Zealand rabbits (age: 28 weeks) were divided into two groups (model group and hydrogen group), each containing 30 rabbits. Osteonecrosis was induced in both groups by methods according to the previously published protocols [6, 7]. Briefly, the rabbits received an intramuscular injection of 20 mg/kg body weight of methylpednisolone acetate (MPSL, Pfizer Pharmaceutical, China) into the right gluteus medius muscle. In addition to MPSL, the hydrogen group also received intraperitoneal injections of molecular hydrogen at 10 ml/kg body weight for seven consecutive days starting from the day of MPSL administration, which was based on the period of greatest susceptibility to steroid-induced oxidative injury suggested by Ichiseki et al. [11, 28].

### Haematological examination

The blood samples were obtained from the auricular artery of each rabbit in a fasting state immediately before injection of MPSL(day 0) and 3, 5, 7, 14 days after the MPSL injection. Blood samples were collected at the same time of the day. The levels of total cholesterol (T-cho; mg/dL), triglycerides (TG; mg/dL) and soluble thrombomodulin (sTM; ug/L) at day 0, 7, 14 and the level of GSH(ug/mL) and MDA(umol/L) at day 0, 3, 5, 7, 14 were determined.

### Histopathology

Two weeks after steroid administration, the rabbits were killed with an overdose of sodium pentobarbital. Bilateral femurs were obtained for histological analyses. For HE staining, bone samples were fixed with 10% neutral buffered formalin for 1 week, decalcified with 10% ethylene diamine tetraacetic acid (EDTA) for 4 weeks, then embedded in paraffin, cut into 4 μm thick sections and stained with hematoxylin-eosin. The bone samples were cut along the coronal plane in the proximal one third and axial plane in the distal part (condyle).

Whole areas of bilateral femoral samples, including the proximal one-thirds and distal condyles, were histopathologically examined for the presence of ON. A positive diagnosis of ON was determined based on the diffuse presence of empty lacunae or pyknotic nuclei of osteocytes within the bone trabeculae, accompanied by surrounding bone marrow cell necrosis or fat cell necrosis [29]. 8 sections were taken from the proximal one-thirds and distal condyles of both femurs of each rabbit. The presence or absence of ON was determined using well established criteria [29, 30]. Briefly, rabbits that had at least one osteonecrotic lesion in the 8 sections were considered as ON+, while those with no osteonecrotic lesions were defined as ON−. Besides, the incidence of ON was calculated as the ratio of the number of rabbits with ON/total number of rabbits used in each group.

During the observation, the average bone marrow fat cell size was calculated using image analysis software (ImageJ 1.32j, NIH, USA). As described in previous reports, the average bone marrow fat cell size was defined as the average of the Feret’s diameter of all bone marrow fat cells in 4 randomly-selected non-necrotic fields (16 fields for 4 dissected parts from each rabbit). Fat cells that had undergone necrosis were excluded from imaging analysis. During the observation, both the judgment of osteonecrosis and the calculation of average fat cell size were performed by two researchers who were blind to the identity of the hydrogen group.

### Ink artery infusion angiography

Two weeks after steroid administration, the rabbits were anesthetized with sodium pentobarbital and ink artery infusion angiography was performed to investigate vascular injury in the femur according to the previously published protocol [31]. After the infusion, the animals were then euthanized and stored at 4 °C for 24 h. Then the bilateral thighbones were dissected and the samples were fixed, decalcified, embedded and cut into 5-μm-thick slices. Ink leakage would be observed if vascular permeability increased which was induced by vascular injury.

### Immunohistochemistry

To determine the level of oxidative injury, the femurs were stained immunohistochemically with anti-8-hydoxy-2-deoxyguanosine (8-OHdG) monoclonal antibody (N45.1) and anti-malondialdehyde (MDA) monoclonal antibody (clone 1 F83) which was major marker of DNA oxidative injury and lipid peroxide respectively [14, 28]. Briefly, sections were immersed in 3% hydrogen peroxide PBS for 10 min, rinsed several times in PBS, pretreated with 10% goat normal antiserum(Vector, Burlingame, California, USA) for 30 min at room temperature. Then, sections were reacted with a primary antibody overnight at 4 °C and incubated with the biotinylated secondary antibody for 30 min, followed by streptavidin peroxidases for 30 min at room temperature. Finally, sections were treated with diaminobenzidine solution, counterstained with haematoxylin and mounted. Sections without primary antibodies processing were used as the negative control. The sections were observed and photographed by the eclipse 50i optical microscope imaging system (Nikon Co., Ltd., Tokyo, Japan).

To quantify DNA oxidation injury and vascular damage of immunohistological results, the 8-OHdG positive area per visual field (PA%) and the MDA positive marrow vessels were calculated by image analysis software (ImageJ 1.32j, NIH, USA). DNA oxidative injury was evaluated by calculating the %PA of 8-OHdG, the percentage of 8-OHdG positive area/total area, in 5 randomly selected visual fields (×200 magnification) [7]. Vascular damage was evaluated by counting the number of MDA positive marrow vessels in 5 randomly-selected fields from the proximal one-third of the femur (×100 magnification) [14].

### TUNEL assays

The terminal deoxynucleotidyl transferase-mediated dUTP nick end labeling (TUNEL) assays were performed by using the TUNEL detection kit (Promega Co., Ltd., Beijing, China) to observe apoptotic osteoblasts and osteocytes according to the manufacturer’s instructions. The cells with brown nuclei were assessed as positive. During the observation of per tissue slide at 200 times magnification, the apoptotic rate was calculated by two blinded researchers. The apoptotic rate was defined as number of TUNEL-positive cells/number of all cells. Besides, morphological changes characteristic of apoptosis were examined carefully to minimize ambiguity regarding the interpretation of positive results.

### Statistical analysis

Categorical data, i.e. incidence of osteonecrosis, was analyzed using Pearson’s chi-square test. All the numerical data were described as mean ± standard deviation. Simple comparisons of numerical data were performed using Student’s *t*-test. To compare the serial changes of GSH and MDA levels in each group, a repeated-measures analysis of variance (ANOVA) was used. To compare the changes in GSH and MDA values between the hydrogen group and the model group, a two-way ANOVA was used. The SPSS 17.0 software was used for analysis. *P* values < 0.05 were considered significant.

## Results

### Histopathological observations

Seven of the 60 rabbits (5 in the model group and 2 in the hydrogen group) died of the inductive protocol throughout the experiment period and were excluded from the evaluation.

**Fig. 1:**
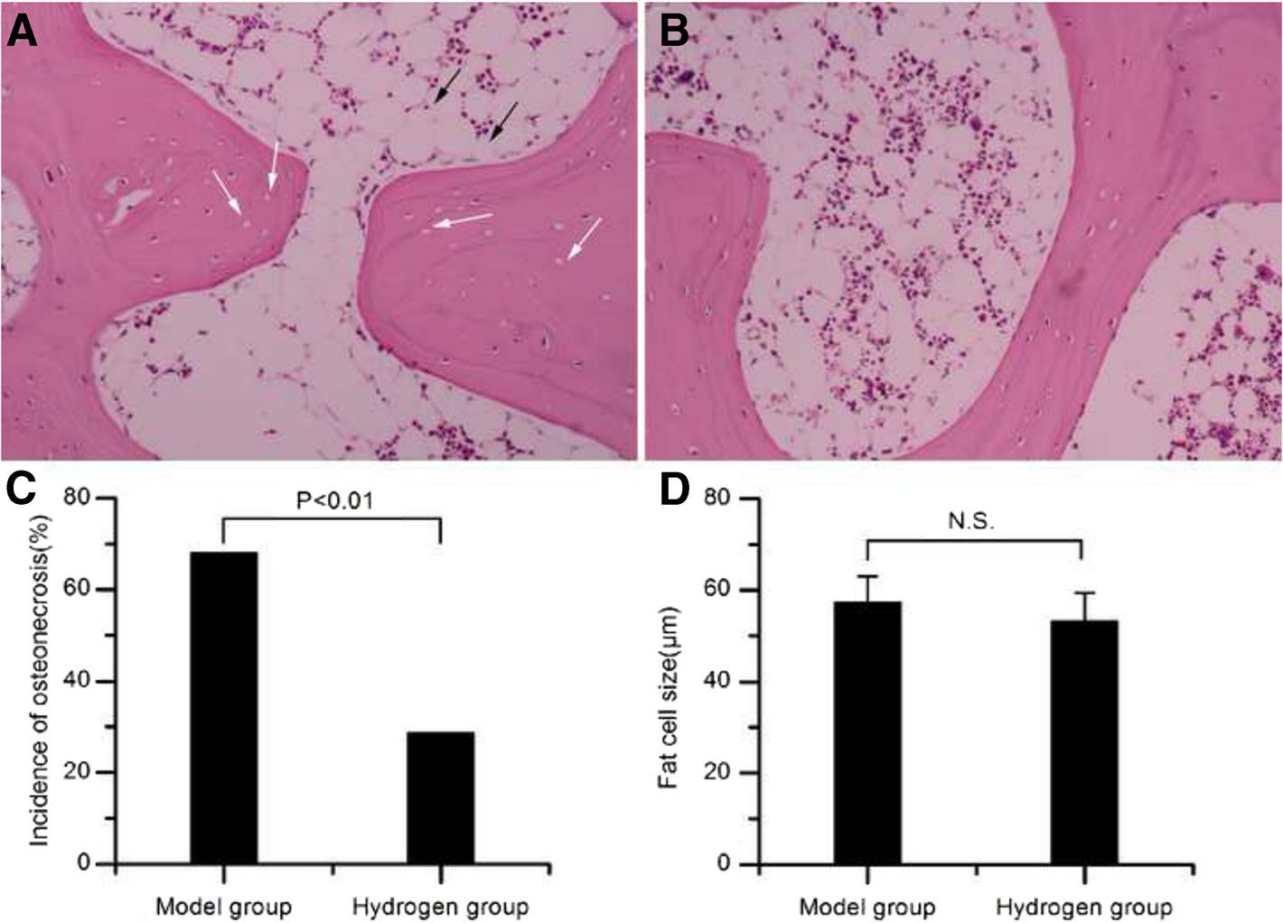
Histological observation. **a** Representative ON+ sample in the model group showed more empty lacunae within the bone trabeculae (indicated by white arrow), surrounded by necrotic medullary haematopoietic cells and necrotic fat cells(indicated by black arrow). **b** Representative ON− sample in the hydrogen group showed less empty lacunae or osteocytes having pycnotic nuclei, surrounded by abundant normal marrow hematopoietic cells. **c** The ON incidence in the hydrogen group was significantly lower than that of the model group (*P <* 0.01). **d** There were no statistical difference about the size of fat cells between the hydrogen group and the model group (*P >* 0.05). N.S., not significant

Osteonecrosis was observed in 17 of the 25 rabbits in the model group and in 8 of 28 rabbits in the hydrogen group (Fig. 1). The incidence of osteonecrosis was significantly lower in the hydrogen group than that in the model group (Pearson’s chi-square test, *P <* 0.01). The size of fat cells was 57.3 ± 5.8 μm in the model group and 53.6 ± 6.2 μm in the hydrogen group (*P >*0.05).

### Hematological findings

**Fig. 2:**
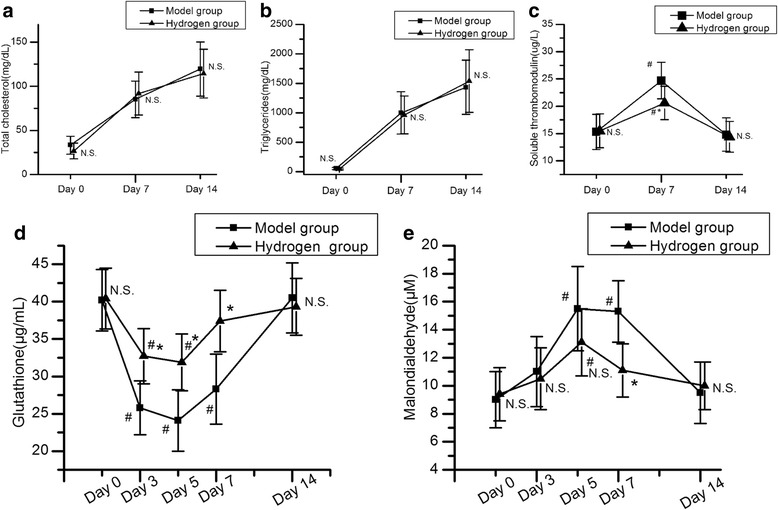
The levels of total cholesterol (**a**), triglycerides (**b**), soluble thrombomodulin (**c**), glutathione (**d**) and malondialdehyde (**e**). ^#^ *P <* 0.05 for comparison with baseline; ^*^ *P <* 0.05 for comparison with the model group. N.S., not significant for comparison with the model group

Hematological data were compared both between different time points in the same group and between the model group and the hydrogen group at the same time points. The levels of total cholesterol and triglycerides in both groups increased during the period from day 7 to day 14 after MPSL administration (*P <* 0.05). However, there was no difference between the two groups at all the same time points (*P >* 0.05) (Fig. 2a and b). The sTM level significantly increased at day 7 in the model group and then declined toward baseline. However, in the hydrogen group, the significant increase of sTM was prevented (Fig. 2c). In the model group, the GSH level was significantly decreased between 3 and 7 days after steroid administration (*P <* 0.05), followed by a tendency to recover by 7–14 days after administration. The MDA level was significantly increased between 5 and 7 days after steroid administration (*P <* 0.05) and appeared to recover by 7–14 days after administration in the model group. Both decreases in GSH and increases in MDA of the hydrogen group were significantly attenuated compared with those in the model group (Fig. 2d and e). In the hydrogen group, the decrease of GSH level was significantly suppressed on days 3 and 5 after MPSL administration (*P <* 0.05), and the increase of MDA level was significantly suppressed on day 7 after MPSL administration (*P <* 0.05).

### Oxidative stress and vascular injury in the proximal femurs

**Fig. 3:**
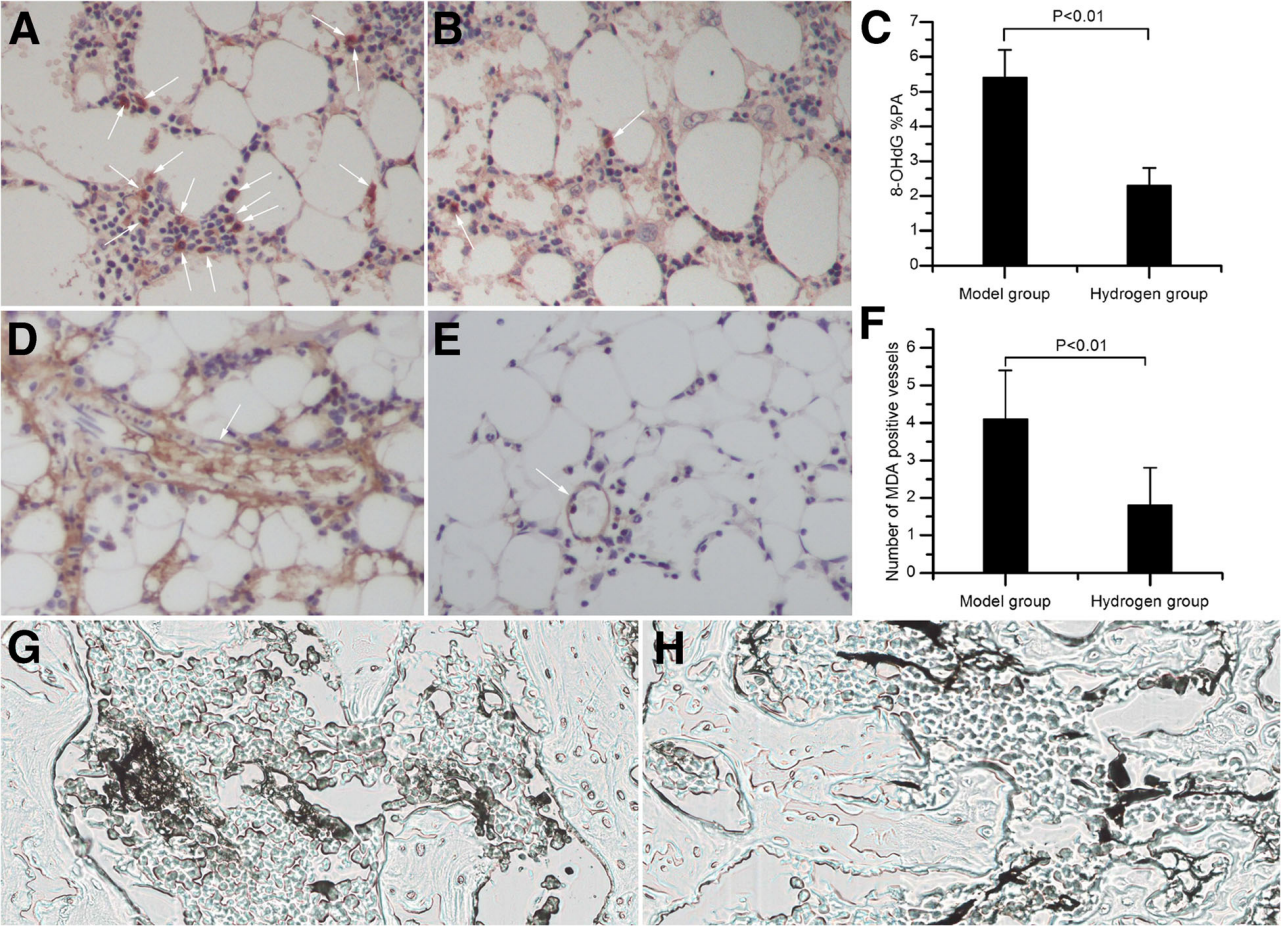
Oxidative stress and vascular injury in the proximal femurs. **a** The model group showed clusters of 8-OHdG-positive haematopoietic cells in marrow. **b** In the hydrogen group, sporadic 8-OHdG-positive haematopoietic cells were found. **c** The hydrogen group exhibited a lower 8-OHdG %PA than the model group (*P <* 0.01). **d** Strong immunoreactivity for MDA was found in marrow vessels and haematopoietic cells of the model group. **e** The hydrogen group exhibited weak immunoreactivity for MDA in marrow vessels and haematopoietic cells. **f** A smaller number of MDA positive vessels was found in the hydrogen group than the model group (*P <* 0.01). **g** Stronger ink leakage was observed in the model group. **h** The hydrogen group showed less ink leakage

In marrow of the model group, clusters of 8-OHdG-positive haematopoietic cells were present (Fig. 3a). However, marrow of the hydrogen group showed only a few, sporadic 8-OHdG-positive haematopoietic cells (Fig. 3b). Furthermore, 8-OHdG %PA was 2.3 ± 0.5% in the hydrogen group (Fig. 3c), significantly lower than 5.4 ± 0.8% in the model group (*P <* 0.01).

In the model group, strong immunoreactivity for MDA was found in marrow vessels and haematopoietic cells (Fig. 3d), whereas weak immunoreactivity for MDA was found in the hydrogen group (Fig. 3e). Furthermore, the number of MDA positive vessels was 4.1 ± 1.9 in the model group (Fig. 3f). In the hydrogen group, a smaller number of MDA positive vessels, 1.8 ± 1.0, was observed (*P <* 0.01).

Stronger ink leakage indicating increased vascular permeability was observed more often in the model group (Fig 3g). In the hydrogen group, less ink leakage was observed (Fig. 3h).

### Apoptosis in the proximal femurs

**Fig. 4:**
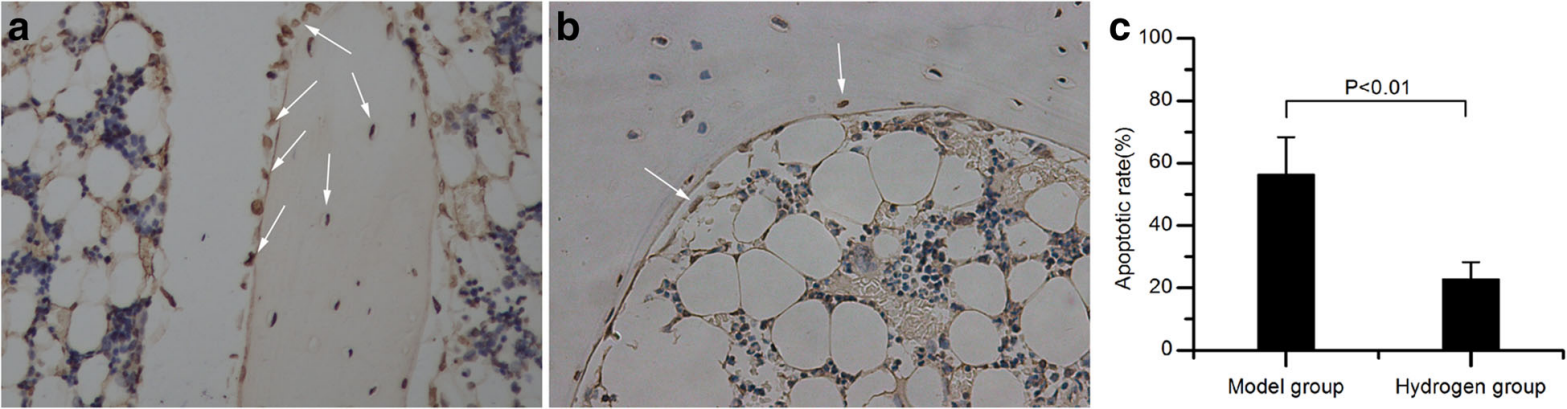
TUNEL apoptosis detection. **a** The model group showed many apoptotic osteoblasts and osteocytes. **b** In the hydrogen group, few TUNEL-positive osteoblasts and osteocytes were found. **c** The apoptotic rate in the hydrogen group was significantly lower than that of the model group (*P <* 0.01)

In the model group, many TUNEL-positive osteoblasts and osteocytes were observed (Fig. 4a). However, few and sporadic TUNEL-positive osteoblasts and osteocytes were observed in hydrogen group (Fig. 4b), which indicated that the administration of molecular hydrogen protected the trabecular bone cells from apoptosis.

## Discussion

Circulatory disturbance [32], vascular endothelial dysfunction [33], enhanced vasoconstriction [3], thrombotic and fibrinolytic disorders [2, 34], impairment of lipid metabolism [35], increased oxidative stress [11, 28] and apoptosis [36] have been suggested to exert important roles in the development of steroid-induced osteonecrosis (ON). Many drugs to inhibit these, including lipid-lowering drugs (Statin) [6, 37], anticoagulants (Enoxaparin) [2, 38, 39], antioxidants (Vitamin E, Lipoic acid, Edaravone) [7, 13, 14, 40] and vasodilation drugs [3] have been reported to effectively prevent or suppress the progression of steroid-induced ON in animals and humans. In the present study, we selected hydrogen gas, which was reported to decrease oxidative injury in various diseases related to oxidative stress and optimize lipid metabolism, as therapy for steroid-induced ON in an established rabbit model. We found that hydrogen therapy significantly decreased the incidence of steroid-induced ON in rabbits to 28% as compared with 68% in the model group, and attenuated both the decreases in plasma levels of GSH and the increases in plasma levels of MDA and sTM compared with those in the model group. Locally in the proximal femurs, DNA oxidative injury, lipid peroxide and vascular endothelial injury, indicated by immunohistochemistry for 8-OHdG, MDA and ink artery infusion angiography respectively, were decreased after hydrogen administration. However, effects of hydrogen on the levels of total cholesterol and triglycerides were not observed.

Disorders of lipid metabolism which resulted in extravascular lipid deposition and elevated intraosseous pressure, played an important role in the etiology of steroid-induced ON [35]. In the present study, disorders of lipid metabolism were evaluated by measuring the levels of total cholesterol and triglycerides as well as fat cell size. Total cholesterol, triglycerides and the fat cell size elevated after steroid administration which was consistent with findings by Kuribayashi et al. [14]. Study by Zong et al. [26] demonstrated that in high-fat diet-fed hamster model, 4-week intraperitoneal injection of hydrogen-saturated saline remarkably decreases plasma total cholesterol and low-density lipoprotein (LDL) cholesterol levels. In an open label, 8-week study on 20 subjects with potential metabolic syndrome, Nakao et al. found administration of hydrogen rich water for 4 weeks increases high density lipoprotein (HDL)-cholesterol and decreases total cholesterol/high-density lipoprotein(HDL)-cholesterol [25]. In addition, study of Kamimura et al. indicated that consuming molecular hydrogen for 3 months suppresses plasma triglyceride level in db/db obesity model mice [27]. In our study, no statistically significant differences about the level of total cholesterol, triglycerides and the fat cell size were found between the model group and the hydrogen group, indicating that disorders of lipid metabolism was not improved by hydrogen during the 2-week duration for observation and some other osteonecrosis-prevention mechanisms exist other than disorders of lipid metabolism. Considering that the beneficial effects of molecular hydrogen on lipid metabolism were based on long-term molecular hydrogen administration, the period of hydrogen treatment and duration for observation should be extended to determine the long-term efficacy of hydrogen treatment on disorders of lipid metabolism in steroid-induced ON.

Increased oxidative stress in the etiology of steroid-induced ON has been well documented [7, 11, 13, 14, 28, 40]. In the present study, we measured the blood levels of GSH and MDA which generally reflect the development of oxidative stress in the body. GSH is an important antioxidant enzyme in vivo and exerts a crucial role in maintaining integrity of cell structure by inhibiting the increase of lipoperoxides and maintaining redox reaction balance. MDA is generated during the hyperoxidation of lipids and can be used as a biochemical indicator of oxidative injury. In our study, we found that GSH levels significantly decreased and MDA levels significantly increased shortly after MPSL administration. The results were in accord with those of Ichiseki et al. [11], Mikami et al. [13], Kuribayashi et al. [14] and Li et al. [7]. In addition, we detected the local expression of 8-OHdG and MDA, which was major marker of DNA oxidative injury and lipid peroxide respectively, in the femurs. Consistent with findings of previous studies [7, 14, 28], the number of both 8-OHdG-positive haematopoietic cells and MDA-positive haematopoietic cells increased. In the prevention group treated with hydrogen, we found that the decrease of GSH and increase of MDA in plasma caused by steroid were significantly attenuated by hydrogen administration. Moreover, the number of both 8-OHdG-positive haematopoietic cells and MDA-positive haematopoietic cells in marrow, and the 8-OHdG positive area per visual field (8-OHdG PA%) decreased in group received hydrogen therapy. This demonstrated that hydrogen could systemically and locally reduce oxidative stress caused by steroid.

Vascular endothelial dysfunction injury was one of the factors that participate in the development of steroid-induced ON [33, 41]. It was reported that lipid peroxide could markedly damage vascular endothelial cells [42] and induce vascular endothelial dysfunction accompanying with oxidative injury in steroid-induced ON [11, 40]. In our study, the results of immunohistochemical staining for MDA and ink artery infusion angiography in the group only received steroid indicated that bone marrow vessels suffered from extensive oxidative injury caused by steroid administration. However, hydrogen administration reduced the number of MDA-positive vessels in marrow compared with the model group, indicating that hydrogen considerably decreased the damage of steroid-induced oxidative stress to endothelial cells. Furthermore, we determined the level of soluble thrombomodulin(sTM) in plasma which is an index of endothelial injury. We found that sTM significantly increased in the model group at day 7 post induction and hydrogen reduced the sTM concentration.

Roles of apoptosis in steroid-induced ON were reported in several studies [15, 43]. Oxidative stress is an important contributor to apoptosis [10]. The results of TUNEL assay in our study demonstrated that molecular hydrogen decreased the number of apoptotic osteoblasts and osteocytes, which was in accord with the decrease of oxidative stress. It indicated that molecular hydrogen might indirectly inhibit apoptosis by suppressing oxidative stress.

Compared with conventional antioxidants, diatomic hydrogen has several potential advantages [17]. First, hydrogen is highly diffusible and it can penetrate biomembranes and diffuse into the mitochondria and nucleus, which are the primary site of ROS generation and DNA damage. Secondly, diatomic hydrogen(H2) could selectively reduce level of hydroxyl radical (•OH) and peroxynitrite (ONOO-) which are the most aggressive ROS, but do not affect the level of H2O2 and the nitric oxide radical (NO•) which play physiological roles in immune defence systems and signal transduction. Thirdly, diatomic hydrogen exerts antioxidative effects with few toxic side effects at effective dosages. Finally, it is cheaper than conventional pharmaceuticals.

One of the limitations of the present study is that we limited the delivery of hydrogen gas to intraperitoneal injection and limited the therapeutic dose of hydrogen to 10 ml/kg. We were unable to determine the optimum way of hydrogen administering and the optimum level of hydrogen. In addition, we did not clarify the further mechanisms at the gene level which might help us to better understand the preventive effects of hydrogen on osteonecrosis. However, it was indicated that genes for oxidoreduction-related proteins were up-regulated in the livers of rats after 4 weeks of drinking hydrogen-rich water [44]. This might partially clarify the antioxidative effects of hydrogen on osteonecrosis. Thus, in the near future, we will conduct additional studies to determine the optimum level of hydrogen that can prevent the development of osteonecrosis. Besides, we will determine the changes of oxidoreduction-related genes in the femurs after hydrogen administration.

## Conclusions

In conclusion, the present results suggest that oxidative stress participated in the development of steroid-induced ON and molecular hydrogen could significantly reduce the incidence of steroid-induced ON possibly by systemically and locally reducing oxidative stress and endothelial injury. This might offer a novel and simple method to prevent the development of steroid-induced ON. Furthermore, the long-term efficacy of molecular hydrogen therapy on disorders of lipid metabolism in steroid-induced ON needs to be explored.

## Abbreviations

8-OHdG: 8-hydoxy-2-deoxyguanosine
EDTA: Ethylene diamine tetraacetic acid
GSH: Glutathione
HDL: High density lipoprotein
HUVECs: Human umbilical vein endothelial cells
LDL: Low-density lipoprotein
LPS: Lipopolysaccharide
MDA: Malondialdehyde
MPSL: Methylpednisolone acetate
ON: Osteonecrosis
ROS: Reactive oxygen species
sTM: Thrombomodulin
T-cho: Total cholesterol
TG: Triglycerides
TUNEL: The terminal deoxynucleotidyl transferase-mediated dUTP nick end labeling
